# Designing and Evaluating an Interactive Multimedia Web-Based Simulation for Developing Nurses’ Competencies in Acute Nursing Care: Randomized Controlled Trial

**DOI:** 10.2196/jmir.3853

**Published:** 2015-01-12

**Authors:** Sok Ying Liaw, Lai Fun Wong, Sally Wai-Chi Chan, Jasmine Tze Yin Ho, Siti Zubaidah Mordiffi, Sophia Bee Leng Ang, Poh Sun Goh, Emily Neo Kim Ang

**Affiliations:** ^1^National University of SingaporeSingaporeSingapore; ^2^The University of NewcastleNewcastleAustralia; ^3^National University HospitalSingaporeSingapore

**Keywords:** acute nursing care, authentic learning, clinical competency, deterioration, multimedia, instructional strategies, simulation, Web-based simulation

## Abstract

**Background:**

Web-based learning is becoming an increasingly important instructional tool in nursing education. Multimedia advancements offer the potential for creating authentic nursing activities for developing nursing competency in clinical practice.

**Objective:**

This study aims to describe the design, development, and evaluation of an interactive multimedia Web-based simulation for developing nurses’ competencies in acute nursing care.

**Methods:**

Authentic nursing activities were developed in a Web-based simulation using a variety of instructional strategies including animation video, multimedia instructional material, virtual patients, and online quizzes. A randomized controlled study was conducted on 67 registered nurses who were recruited from the general ward units of an acute care tertiary hospital. Following a baseline evaluation of all participants’ clinical performance in a simulated clinical setting, the experimental group received 3 hours of Web-based simulation and completed a survey to evaluate their perceptions of the program. All participants were re-tested for their clinical performances using a validated tool.

**Results:**

The clinical performance posttest scores of the experimental group improved significantly (*P*<.001) from the pretest scores after the Web-based simulation. In addition, compared to the control group, the experimental group had significantly higher clinical performance posttest scores (*P*<.001) after controlling the pretest scores. The participants from the experimental group were satisfied with their learning experience and gave positive ratings for the quality of the Web-based simulation. Themes emerging from the comments about the most valuable aspects of the Web-based simulation include relevance to practice, instructional strategies, and fostering problem solving.

**Conclusions:**

Engaging in authentic nursing activities using interactive multimedia Web-based simulation can enhance nurses’ competencies in acute care. Web-based simulations provide a promising educational tool in institutions where large groups of nurses need to be trained in acute nursing care and accessibility to repetitive training is essential for achieving long-term retention of clinical competency.

## Introduction

Acquiring nursing competencies in assessing and managing acutely ill patients has been identified as a crucial learning goal in the acute care hospital to reduce the occurrence of adverse events such as cardiopulmonary arrest [1,2]. Previous studies have shown the benefits of using mannequin-based simulation in acute nursing care training [3,4]. As simulation places the learners in a realistic situation, it is considered an authentic learning activity to prepare learners for their real-life work. Lebow described authentic activity as “experiences of personal relevance that permit learners to practice skills in environments similar to those in which the skills will be used” [5]. The authentic learning activities embedded in simulation enable the learners to develop knowledge, skills, and attitudes in an integrative whole, which facilitates the transfer to future real-life work settings [6]. However, developing and implementing an authentic learning environment using simulation is often limited by the availability of simulation facilities, the requirement for trained simulation instructors, and the small numbers of learners involved in the training at one time [7]. Given the resource-intensive nature of simulation, it would be a challenge for institutions that need to train large groups of nurses. Nonetheless, such limitations could be eased with the development of Web-based learning (WBL) technology.

With technological advancements, WBL can provide an authentic learning context that responds to nurses’ needs and experiences in clinical practice. WBL is endorsed as an essential educational tool for lifelong learning and professional development [8]. Previous studies did not show any differences between WBL and face-to-face learning in the acquisition of knowledge, skills, and satisfaction [9]. Additionally, nurses perceived that WBL was suited to meeting their working conditions and needs including conflicting work schedules and intensive workload [10].

Advances in multimedia technology make the design of authentic learning activities increasingly feasible in WBL. Multimedia refers to technology for presenting visual and verbal material [11]. Instructional designers can use media such as text, graphics, audio, and video to simulate real-world environments where learners can perform authentic tasks. In addition, multimedia technology makes it possible for situating simulations in WBL (known as Web-based simulation). One approach is for learners to play roles in goal-based scenarios driven by assessment [12]. Web-based simulations using virtual patients are now popular in health care education for simulating authentic clinical experiences [13].

A review of literature between 2000 and 2014 identified 18 Web-based simulation programs in nursing education with most involving teaching an aspect of procedural patient care and others on interpersonal communication skills or technique skills for equipment use. Descriptive studies reported high levels of acceptance of Web-based simulations by nursing students [14]. Using experimental studies, some of the Web-based simulations were tested by comparing them with mannequin-based simulation. These studies revealed that Web-based simulations are at least as good as, if not better than, mannequin-based simulations in teaching the necessary skills and knowledge to develop competent nursing students [15,16]. As the existing Web-based simulations have a predominant focus on pre-registered health professional education [14], further study is recommended to explore the use of Web-based simulation in continuing health professional education [17].

It appears Web-based simulation is more cost-effective than mannequin-based simulation for training large groups of hospital nurses. The successful implementation of mannequin-based simulation for undergraduate nurses in acute nursing care has prompted us to develop an alternative authentic learning strategy in continuing nursing education of hospital nurses [18]. The use of WBL is still fairly new in the field of acute nursing education. WBL instructional design methods vary considerably, and research is needed to find the most effective method [19]. Thus, the aim of this study is to describe the design, development, and evaluation of an interactive multimedia Web-based simulation for developing nurses’ competencies in acute nursing care.

## Methods

### Overview

The instructional design model developed by Smith and Ragan [20] was used to guide the design, development, and evaluation of the interactive multimedia Web-based simulation. The model focuses on three key activities: analysis, strategy, and evaluation.

### Analysis

#### Learners

The targeted learners were registered nurses (RNs) who have acquired either a diploma or baccalaureate nursing degree. In caring for acutely ill patients, RNs play an important role in recognizing patient deterioration, conveying their assessments to health care staff, and providing timely and appropriate intervention before the arrival of appropriate help.

#### Learning Objectives

With the analysis of nurses’ roles in caring for acutely ill patients, three specific learning objectives were identified: (1) understanding the underlying physiological signs of patient deterioration, (2) recognizing and managing deteriorating patients, and (3) communicating effectively about patient deterioration.

#### Learning Tasks

An important learning task to help nurses recognize the early signs of patient deterioration involves understanding the physiological compensatory mechanism and pathophysiology underpinning changes in vital signs. Here, the “ABCDE” (Airway, Breathing, Circulation, Disability and Expose/Examine) and “ISBAR” (Identity, Situation, Background, Assessment and Recommendation) mnemonics provide frameworks to guide nurses in assessing, managing, and reporting on patients’ deterioration. Specifically, the ABCDE mnemonic encourages a systematic approach to recognizing and responding to deteriorating ward patients. The ISBAR mnemonic provides a structured communication tool for nurses to report on patients’ deterioration. A panel of national experts developed an evaluation tool that assessed the suitability of tasks to be performed by ward nurses in response to patient deterioration. The learning tasks were adapted from this evaluation tool. An international panel of nursing and medical experts further validated the evaluation tool [21].

#### Learning Context

An educational program using simulation was implemented in a Singapore acute care hospital to support early recognition of and intervention in patient deterioration. Despite the benefits of simulation training, there are logistical and resource issues in implementing this kind of training for a large group of nurses. Nurse educators and hospital administrators highly recommended an alternative effective learning strategy that can overcome these difficulties and be accessible to all the hospital nurses.

### Strategy

#### Overview

**Figure 1:**
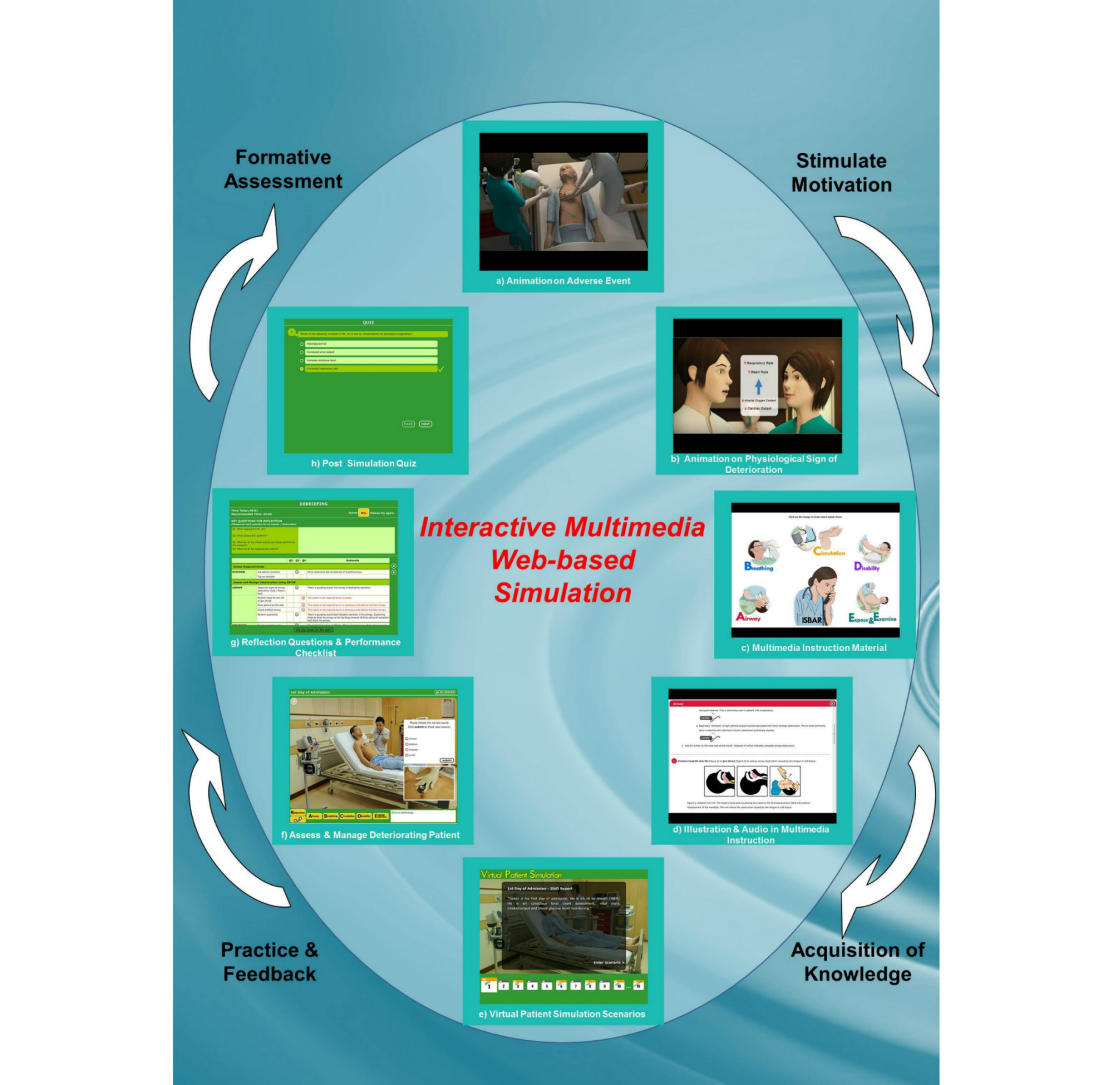
Instructional activities in Web-based simulation.

The learning objectives guided the development of the learning content. As shown in Figure 1, the content delivery sequence includes four key events: (1) stimulate motivation, (2) acquisition of knowledge, (3) practice and feedback, and (4) formative assessment (see Multimedia Appendix 1 for a video). These are facilitated through a variety of instructional activities including animation video, multimedia instructional material, virtual patient simulation, and online quizzes.

#### Stimulate Motivation

To arouse the learners’ motivation to learn, a narrative about a patient experiencing an adverse event (cardiopulmonary arrest) is presented through an animation video. The narrative highlights that such an event can be prevented through the early recognition of patient deterioration (Figure 1a).

#### Acquisition of Knowledge

Information about the underlying physiological signs of patient deterioration is presented in an animation showing two nurses in conversation (Figure 1b). Onscreen text and illustrations are used to explain the tasks involved in assessing, managing, and reporting on the acutely ill patient. These performance steps are organized using evidence-based mnemonic strategies (Figure 1c). The ABCDE mnemonic is employed to help organize the steps of assessing and managing the deteriorating patient, while the ISBAR mnemonic is used to show the steps for reporting on the deteriorating patient. Rationales from evidence-based practice on these steps are elaborated to help learners understand why these steps are important as well as how to understand them. Illustrations and audio lung sounds are employed to assist users in understanding the facts (Figure 1d).

#### Practice and Feedback

Virtual patient simulation is designed to give learners the opportunity to gain practical experience related to the information being learned. Common deteriorating conditions (ie, airway obstruction, breathlessness, hypotension, tachycardia, oliguria, altered consciousness, and abnormal temperatures) associated with acute medical conditions are embedded in five simulation scenarios pertaining to events occurring on different days of the virtual patient’s admission (Figure 1e). In each scenario, the learner emulates the role of a nurse assessing and managing the deteriorating virtual patient by selecting actions from the ABCDE control menus. Immediate feedback, including physiological changes, is programmed into the system to respond to the learner’s actions. ISBAR control menus are used to aid learners in reporting on the virtual patient’s deterioration (Figure 1f). At the end of each scenario, the learners are asked debriefing questions to help them reflect on their experiences. In addition, using an evaluation tool, the learners receive feedback on the appropriate and inappropriate actions taken in the simulation scenario (Figure 1g).

#### Formative Assessment

Formative assessment is seamlessly integrated with the simulation activity. The learners’ performances in each scenario are scored using a validated evaluation tool. Multiple-choice questions related to each simulation scenario are constructed to assess learners’ knowledge of the subject content (Figure 1h).

### Evaluation

#### Design and Sample

A prospective, randomized controlled trial (RCT) with a pretest-posttest design was conducted from November to December 2013. The National University of Singapore Institutional Human Research Ethics Board approved the study. A total of 70 registered nurses, with less than 5 years of work experience and who were working in general ward units, were recruited from an acute care tertiary hospital in Singapore. A sample of 60 participants (30 from each group) was considered adequate to achieve statistical power at the 5% level of significance [20]. Allowing for an attrition rate of 10%, a total of 35 participants were recruited to each arm. They were randomly assigned to an experiment group (N=35) and a control group (N=35) using a computerized random number generator. However, three participants from the control group did not turn up for the study, leaving that group with 32 participants. All participants were given a participant information sheet that explained the purpose of the study.

#### Procedure

**Figure 2:**
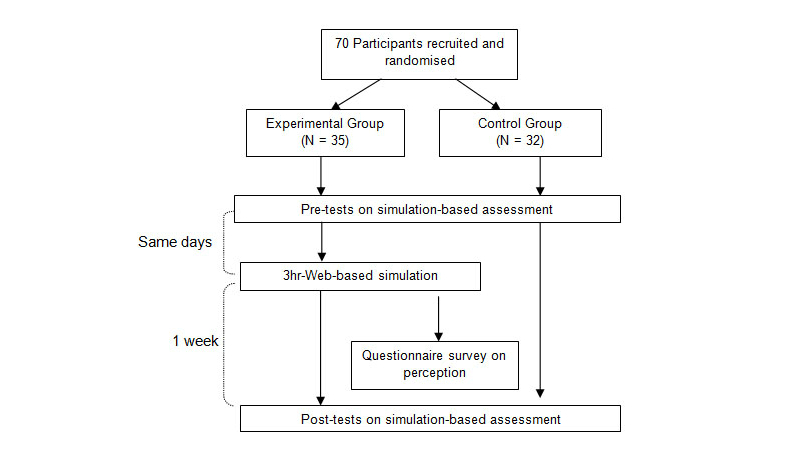
Flow of data collection.

Figure 2 depicts the flow of the study procedure. All participants completed a questionnaire on demographic information. The participants undertook a performance pretest consisting of a simulation-based assessment that took place at the university simulation laboratory. They hid their identities from the raters by putting on caps, gowns, and masks. Following an orientation to the simulation set-up, each participant was given a test scenario with the patient simulator displaying signs and symptoms of clinical deterioration. Each participant was given 15 minutes to assess and manage the deteriorating patient simulator. The entire simulation process was recorded on video. Immediately after the performance pretest, the participants in the experimental group were brought individually into a room with a computer set-up to undertake a 3-hour Web-based simulation. After completing the learning, the participants were asked to complete a questionnaire to evaluate their perception of the Web-based simulation. About a week after the pretests and intervention, the participants from both groups were scheduled to undertake the performance posttests on simulation-based assessment individually, which were similar to the performance pretests.

#### Data Collection and Instruments

Research staff observed and rated the recorded performance using a validated tool known as the RAPIDS (Rescuing a Patient in Deteriorating Situations) tool. The psychometric properties of the RAPIDS tool, including content and construct validity, and interrater reliability were tested and supported in a previous study [21]. Of the recorded videos, 30% (20/67) were randomly selected in this study for rerating by another member of the research staff. There was excellent interrater reliability between the 2 raters, with a high intraclass correlation coefficient (ICC) of .98 (95% CI 0.95-0.99).

An 18-item scale with four subscales (system quality, information quality, user satisfaction, and net benefit) was administered to explore the participants’ perception of the Web-based simulation. This scale was adapted and modified from the e-learning systems success (ELSS) scale [22]. The study obtained a high internal consistency of this scale with Cronbach alpha at .97. Two questions were added to allow for open-ended comments.

#### Data Analysis

Descriptive statistics were computed for the demographic variable and participants’ perceptions of the Web-based simulation. Differences in demographic characteristics between the two groups were examined by a chi-square test and *t* test. Interrater reliability was assessed using ICC. Changes between baseline and posttest scores were determined by a paired *t* test. Analysis of covariance (ANCOVA) was performed to evaluate the posttest scores between groups with baseline scores as a covariate. The comments for the two open-ended questions were coded and analyzed for recurring themes.

## Results

Most of the participants were female (97%) and Chinese (62.7%), with an average age of 25.58 years (SD 3.19). Demographic characteristics including gender (*P*=1.00), ethnicity (*P*=.98), age (*P*=.12), years of work experience (*P*=.10), and highest qualification (*P*=.40) were similar for both groups of participants. This supported the randomization and homogeneity of the participants between the groups.

**Figure 3:**
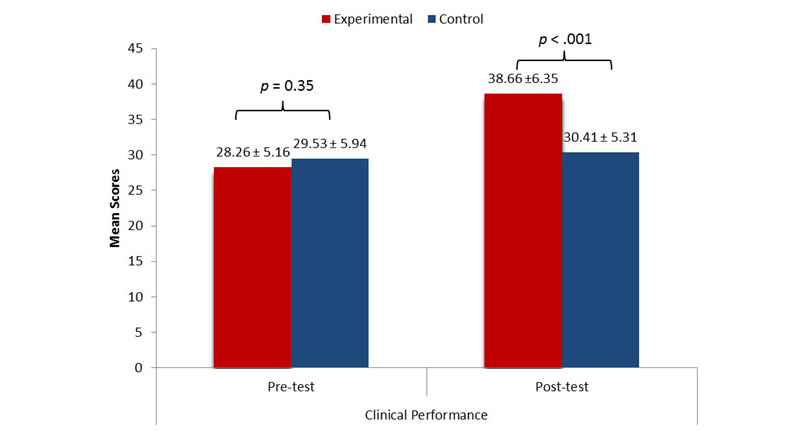
Pretests and posttests scores on clinical performance.

Figure 3 shows that the clinical performance pretest scores did not differ significantly between the experimental and control groups. Within the groups, the clinical performance posttest scores for the experimental group were significantly higher than the pretest scores (*P*<.001). No significant difference was found between the clinical performance pretest and posttest scores for the control group. Between-group comparisons found significantly higher clinical performance posttest scores (*P*<.001) for the experimental group than the control group after controlling the pretest scores.

The mean scores from the participants’ rating (experimental group) on the 7-point scale indicated that that they were highly satisfied with the Web-based simulation (mean 6.00, SD 0.79), positive about the quality of the system (mean 6.04, SD 0.68) and information (mean 5.13, SD 0.62), and thought highly of the net benefits (mean 6.11, SD 0.71) of the program.

Three themes with categories emerged from the written comments on the most valuable aspects of the Web-based simulation: (1) relevance to practice as it provides useful information (ie, “provide specific information which is very useful for us nurses”) and allows application in a practice setting (ie, “can apply in real-life situation”), (2) instructional strategies including animation, e-simulation, and mnemonics (ie, “ABCDE and ISBAR” make it simple and clear to understand”) fostering problem solving through knowledge gained (ie, “understand the signs and symptoms of early deterioration”), and (3) the use of critical thinking (ie, “utilizes critical thinking to choose appropriate nursing assessment and intervention”). Three main themes emerged on ways to improve the WBL program: more variation, more information, and technical features.

## Discussion

### Principal Findings

This randomized controlled study provided evidence of the effectiveness of a Web-based simulation in improving hospital nurses’ acute care competencies. The development of these competencies required the hospital nurses to apply and integrate a broad range of knowledge, skills, and attitudes. In this study, we developed a Web-based simulation that provided opportunities for the learners to work on authentic nursing activities in an authentic learning environment. Authentic activities are tasks learners perform in solving real-life problems [23]. An authentic environment resembling a hospital ward was developed to enhance the learning experience. By providing a context that reflects the way knowledge and skills will be used in actual life, the authentic learning environment stimulates the learners to develop competencies relevant to their working lives [24].

Apart from real-world relevance, a variety of instructional strategies that were drawn from instructional design models were incorporated into the Web-based simulation to develop the nurses’ clinical competencies. The nurses valued the instructional strategies as well as the problem-solving skills fostered through the learning process. The learning process applied theories from a range of fields including cognitive overload, motivation, and multimedia learning, The process began by stimulating the learners’ motivation followed by the acquisition of factual knowledge of the learning tasks using multimedia such as animation video, texts, audio, and illustrations. The importance of incorporating a variety of activities to gain and sustain learner attention was identified as a key element in a motivation model of instructional design [25]. The acquisition of factual knowledge was supported by the use of mnemonics (ABCDE and ISBAR) to assist the learners with mental models and cognitive aids [26].

Drawing on the experiential learning theory [27], the virtual patients in the Web-based simulation provided opportunities for the learners to apply their knowledge through practice in multiple scenarios. Each scenario involved the application of knowledge to problem solve the deteriorating virtual patient by collecting and integrating patient assessment data to arrive at a nursing diagnosis and provide nursing interventions. The use of virtual patients in professional health education has been attributed to the development of clinical reasoning [28-30]. The outcome of this study sheds light on the effectiveness of applying several instructional design strategies based on tested theories in the acquisition of clinical competency. This calls on educators to apply these strategies in the design and implementation of e-learning.

Our findings are consistent with several previous studies that demonstrated the effectiveness of WBL in improving learning outcomes when compared with no intervention [9,31]. Although evidence from previous studies did not indicate the superiority of WBL over other non-computer training methods, it has been shown to be at least as good as, if not better than, instructor-led methods [32] including mannequin-based simulation [15]. Our study demonstrated that, with advances in multimedia, the features of mannequin-based simulation can be built into the Web-based simulation through the use of virtual patients. A systematic review identified that the use of instructional design features including interactivity, practice exercises, repetition, and feedback can favorably influence learning outcomes [32]. These features, which are also used in mannequin-based simulation, were employed in the design of the virtual patients. Learners had the opportunity to engage with the virtual patient in multiple and varied scenarios to enable them to gain repetitive practice. The ability to give various types of feedback, including verification (eg, verifying the learners’ actions) and elaborated feedback (eg, elaborate explanation-style feedback), was incorporated into the design of virtual patients. “Deliberate practice with multiple examples and feedback” has been highlighted as the best approach to the acquisition of clinical competency [33]. Hence, it is evident that the virtual patient in this Web-based simulation has a significant impact on improving the learners’ clinical competency.

Participants’ positive evaluation of the Web-based simulation, along with improvement in their learning outcomes, is consistent with research findings relating to the efficacy of mannequin-based simulation in acute nursing care training [2,3]. However, compared with mannequin-based simulation, Web-based simulation would be a viable option in institutions where large numbers of learners have to be trained. Given the requirements for simulation facilities, facilitators, and small-group learning, mannequin-based simulation has constraints in providing scalable and sustainable training [34]. In contrast, Web-based simulation allows repetitive training, which is absolutely essential for achieving long-term retention of clinical competency [7]. Although Web-based simulation has fewer constraints than mannequin-based simulation, it should not be seen as a substitute for mannequin-based simulation. Unlike Web-based simulation, mannequin-based simulation provides “hands on” kinesthetic learning in a realistic and dynamic situation, which is considered to prepare nurses well for their real-life work. Both are different learning strategies but could be used as complementary learning tools, forming part of a blended-learning strategy to optimize clinical competency gains [7,35].

### Strengths and Limitations

A rigorous research methodology, RCT, was used to evaluate the learning outcomes. However, the quality of the evidence could be limited by the no-intervention control group. Given that this study is looking at the development of a new WBL program for hospital nurses, the no-intervention controlled study is still considered valuable in the early stages of an innovation. The survey evaluation, particularly the qualitative data, shed light on the value of the program and ways to enhance it. Comparisons of other WBL instructional designs could be conducted in future studies to enhance the program in the future. We tested the learners’ clinical performances using a validated and tested instrument. However, due to logistic constraints, we did not measure the long-term retention of clinical performances, which can deteriorate over time. Future studies could evaluate this competence over a longer period of time. The present study may have shown the effectiveness of Web-based simulation in the context of transferring learning from the Web-based to the simulated environment. Moving forward, future studies could determine the higher-order outcomes of the program on actual clinical practice by evaluating nurse behaviors in practice and their effects on patient care.

### Conclusions

Changes in health care delivery mean that nurse educators need to ensure the competency of all hospital nurses in acute nursing care for optimal patient care outcomes. In this study, we described how Web-based simulation can be effectively implemented to create an authentic nursing activity for developing hospital nurses’ competencies in acute nursing care. A broad array of instructional strategies based on tested theories, including animation video, multimedia instructional material, virtual patient, and online quizzes, were incorporated into the interactive multimedia Web-based simulation. Using a randomized controlled study, we demonstrated the effectiveness of the Web-based simulation in developing nurses’ clinical competencies. This study provides evidence for the acceptance of this Web-based simulation for continuing nursing education among hospital nurses. Nurse educators can use Web-based learning technology to improve the efficiency and effectiveness of educational intervention in the face of pedagogical challenges, especially those posed by mannequin-based simulation. More research is needed to inform the effective use of Web-based simulation by aligning and optimizing its use with other educational technologies, as part of a blended-learning strategy.

## Multimedia Appendix 1

Multimedia interactive Web-based simulation.

## Multimedia Appendix 2

CONSORT-EHEALTH checklist V1.6.2 [36].

## Abbreviations

ABDCDE: Airway, Breathing, Circulation, Disability, and Expose/Examine
ANCOVA: analysis of covariance
ELSS: e-learning systems success
ISBAR: Identity, Situation, Background, Assessment, and Recommendation
RAPIDS: Rescuing a Patient in Deteriorating Situations
RCT: randomized controlled trial
RN: registered nurse
WBL: Web-based learning
