# Correction: Designing and Evaluating an Interactive Multimedia Web-Based Simulation for Developing Nurses’ Competencies in Acute Nursing Care: Randomized Controlled Trial

**DOI:** 10.2196/jmir.4419

**Published:** 2015-03-23

**Authors:** Sok Ying Liaw, Lai Fun Wong, Sally Wai-Chi Chan, Jasmine Tze Yin Ho, Siti Zubaidah Mordiffi, Sophia Bee Leng Ang, Poh Sun Goh, Emily Neo Kim Ang

**Affiliations:** ^1^ National University of Singapore Singapore Singapore; ^2^ The University of Newcastle Newcastle Australia; ^3^ National University Hospital Singapore Singapore

The authors of “Designing and Evaluating an Interactive Multimedia Web-Based Simulation for Developing Nurses Competencies in Acute Nursing Care: Randomized Controlled Trial” (http://www.jmir.org/2015/1/e5/) have overlooked an error in the Methods section during the submission and proofreading process. The phrase “a minimum of 5 years of work experience” should have been “less than 5 years of work experience”. This error has been corrected in the online version of the paper on the JMIR website on March 23, 2015, together with publishing this correction notice. A correction notice has been sent to PubMed and the correct full-text has been resubmitted to Pubmed Central and other full-text repositories.

