# Supplementary material for: Designing and Evaluating an Interactive Multimedia Web-Based Simulation for Developing Nurses’ Competencies in Acute Nursing Care: Randomized Controlled Trial
Source: J Med Internet Res. 2015 Jan 12;17(1):e5. doi: 10.2196/jmir.3853 (PMC4319078; doi:10.2196/jmir.3853)
Supplement: Supplementary file 2 [file jmir_v17i1e5_app2.pdf]

CONSORT-EHEALTH Checklist V1.6.2 Report

(based on CONSORT-EHEALTH V1.6), available at [http://tinyurl.com/consort-ehealth-v1-6].

3853

Date completed

11/29/2014 3:31:06

by

Sok Ying Liaw

Designing and evaluating an interactive multimedia web-based simulation for developing hospital nurses' competencies in acute nursing care: Randomized Controlled Trial

TITLE

1a-i) Identify the mode of delivery in the title

Designing and evaluating an interactive multimedia web-based simulation for developing hospital nurses' competencies in acute nursing care: Randomized Controlled Trial

1a-ii) Non-web-based components or important co-interventions in title

"developing hospital nurses' competencies in acute nursing care"

1a-iii) Primary condition or target group in the title

Designing and evaluating an interactive multimedia web-based simulation for developing hospital nurses' competencies in acute nursing care: Randomized Controlled Trial

ABSTRACT

1b-i) Key features/functionalities/components of the intervention and comparator in the METHODS section of the ABSTRACT

"Authentic nursing activities were developed in a web-based simulation using a variety of instructional strategies including animation video, multimedia instructional material, virtual patients, and online quizzes."

1b-ii) Level of human involvement in the METHODS section of the ABSTRACT

"A randomized controlled study was conducted on 67 registered nurses who were recruited from the general ward units of an acute care tertiary hospital. Following a baseline evaluation of all participants' clinical performance in a simulated clinical setting, the experimental group received three hours of web-based simulation and completed a survey to evaluate their perceptions of the program. All participants were re-tested for their clinical performances using validated tool."

1b-iii) Open vs. closed, web-based (self-assessment) vs. face-to-face assessments in the METHODS section of the ABSTRACT

"A randomized controlled study was conducted on 67 registered nurses who were recruited from the general ward units of an acute care tertiary hospital. Following a baseline evaluation of all participants' clinical performance in a simulated clinical setting, the experimental group received three hours of web-based simulation and completed a survey to evaluate their perceptions of the program. All participants were re-tested for their clinical performances using validated tool."

1b-iv) RESULTS section in abstract must contain use data

"A randomized controlled study was conducted on 67 registered nurses who were recruited from the general ward units of an acute care tertiary hospitalA randomized controlled study was conducted on 67 registered nurses who were recruited from the general ward units of an acute care tertiary hospital"

1b-v) CONCLUSIONS/DISCUSSION in abstract for negative trials

Not applicable as trial was not negative

INTRODUCTION

2a-i) Problem and the type of system/solution

"The use of WBL is still fairly new in the field of acute nursing education. WBL instructional design methods vary considerably and research is needed to find the most effective method [19]. Thus, the aim of this study is to describe the design, development and evaluation of an interactive multimedia web-based simulation for developing nurses' competencies in acute nursing care."

2a-ii) Scientific background, rationale: What is known about the (type of) system

"Using experimental studies, some of the web-based simulations were tested by comparing with mannequin-based simulation. These studies revealed that web-based simulations are at least as good as, if not better than, mannequin-based simulations in teaching the necessary skills and knowledge to develop competent nursing students [15, 16]. As the existing web-based simulations have a predominant focus on pre-registered health professional education [14], further study is recommended to explore the use of web-based simulation in continuing health professional education [17]."

METHODS

3a) CONSORT: Description of trial design (such as parallel, factorial) including allocation ratio

"the aim of this study is to describe the design, development and evaluation of an interactive multimedia web-based simulation for developing nurses' competencies in acute nursing care."

3b) CONSORT: Important changes to methods after trial commencement (such as eligibility criteria), with reasons

No changes after research commencement

3b-i) Bug fixes, Downtimes, Content Changes

No bug fixes or unexpected events

4a) CONSORT: Eligibility criteria for participants

A total of 70 registered nurses, with a minimum of five years of working experience and who were working in general ward units, were recruited from an acute care tertiary hospital in Singapore

4a-i) Computer / Internet literacy

Not applicable as study samples are professional nurses who are expected to be computer / internet literate

4a-ii) Open vs. closed, web-based vs. face-to-face assessments:

Web-based simulation vs no intervention

"A total of 70 registered nurses, with a minimum of five years of working experience and who were working in general ward units, were recruited from an acute care tertiary hospital in Singapore"

4a-iii) Information giving during recruitment

All participants were given a participant information sheet that explained the purpose of the study.

4b) CONSORT: Settings and locations where the data were collected

"The participants undertook a performance pre-test consisting of a simulation-based assessment which took place at the university simulation laboratory"

4b-i) Report if outcomes were (self-)assessed through online questionnaires

Not applicable as no online self-assessment was used

4b-ii) Report how institutional affiliations are displayed
Not applicable as all potential participants are recruited from the same hospital
5) CONSORT: Describe the interventions for each group with sufficient details to allow replication, including how and when they were actually administered
5-i) Mention names, credential, affiliations of the developers, sponsors, and owners
"The first author is the owner and developer of the software"
5-ii) Describe the history/development process
"The successful implementation of mannequin-based simulation for undergraduate nurses in acute nursing care has prompted us to develop an alternative authentic learning strategy in continuing nursing education of hospital nursesThe successful implementation of mannequin-based simulation for undergraduate nurses in acute nursing care has prompted us to develop an alternative authentic learning strategy in continuing nursing education of hospital nurses"
5-iii) Revisions and updating
Not applicable as there is no changes done to the intervention during the evaluation process.
5-iv) Quality assurance methods
"An important learning task to help nurses recognize the early signs of patient deterioration involves understanding the physiological compensatory mechanism and pathophysiology underpinning changes in vital signs. Here, the “ABCDE” (Airway, Breathing, Circulation, Disability and Expose/Examine) and “ISBAR” (Identity, Situation, Background, Assessment and Recommendation) mnemonics provide frameworks guide nurses in assessing, managing and reporting on patients' deterioration. Specifically, the “ABCDE” mnemonic encourages a systematic approach to recognizing and responding to deteriorating ward patients. The “ISBAR” mnemonic provides a structured communication tool for nurses to report on patients' deterioration. A panel of national experts developed an evaluation tool that assessed the suitability of tasks to be performed by ward nurses in response to patient deterioration. The learning tasks were adapted from this evaluation tool. An international panel of nursing and medical experts further validated the evaluation tool [21]."
5-v) Ensure replicability by publishing the source code, and/or providing screenshots/screen-capture video, and/or providing flowcharts of the algorithms used
Screenshots are provides as figure. Screen-capture video are included as supplementary file
5-vi) Digital preservation
Screenshots are provides as figure. Screen-capture video are included as supplementary file
5-vii) Access
"the participants in the experimental group were brought individually into a room with a computer setup to undertake a 3-hour web-based simulation"
5-viii) Mode of delivery, features/functionalities/components of the intervention and comparator, and the theoretical framework
"The learning objectives guided the development of the learning content. As shown in Figure 1, the contents delivery sequence includes four key events: 1) stimulate motivation; 2) acquisition of knowledge; 3) practice and feedback; 4) formative assessment. These are facilitated through a variety of instructional activities including animation video, multimedia instructional material, virtual patient simulation, and online quizzes."

5-ix) Describe use parameters
"the participants in the experimental group were brought individually into a room with a computer setup to undertake a 3-hour web-based simulation"
5-x) Clarify the level of human involvement
"the participants in the experimental group were brought individually into a room with a computer setup to undertake a 3-hour web-based simulation"
5-xi) Report any prompts/reminders used
No applicable as no prompts or reminders were used
5-xii) Describe any co-interventions (incl. training/support)
Not applicable as the web-based learning is a standalone intervention
6a) CONSORT: Completely defined pre-specified primary and secondary outcome measures, including how and when they were assessed
"All participants completed a questionnaire on demographic information. The participants undertook a performance pre-test consisting of a simulation-based assessment which took place at the university simulation laboratory. They hid their identities from the raters by putting on caps, gowns and masks. Following an orientation to the simulation set-up, each participant was given a test scenario with the patient simulator displaying signs and symptoms of clinical deterioration. Each participant was given 15 minutes to assess and manage the deteriorating patient simulator. The entire simulation process was recorded on video. Immediately after the performance pre-test, the participants in the experimental group were brought individually into a room with a computer setup to undertake a 3-hour web-based simulation. After completing the learning, the participants were asked to complete a questionnaire to evaluate their perception of the web-based simulation. About a week after the pre-tests and intervention, the participants from both groups were scheduled to undertake the performance post-tests on simulation-based assessment individually which were similar to the performance pre-tests."
6a-i) Online questionnaires: describe if they were validated for online use and apply CHERRIES items to describe how the questionnaires were designed/deployed
Not applicable as no online questionnaires was used
6a-ii) Describe whether and how “use” (including intensity of use/dosage) was defined/measured/monitored
Not applicable
6a-iii) Describe whether, how, and when qualitative feedback from participants was obtained
"After completing the learning, the participants were asked to complete a questionnaire to evaluate their perception of the web-based simulation"
6b) CONSORT: Any changes to trial outcomes after the trial commenced, with reasons
Not applicable as no changes occurred after the research commenced
7a) CONSORT: How sample size was determined
7a-i) Describe whether and how expected attrition was taken into account when calculating the sample size
"A sample of 60 participants (30 from each group) was considered adequate to achieve statistical power at the 5% level of significance [20]. Allowing for an attrition rate of 10%, a total of 35 participants were recruited to each arm."
7b) CONSORT: When applicable, explanation of any interim analyses and stopping guidelines
No applicable as no interim analysis was conducted and no stopping guidelines were defined.
8a) CONSORT: Method used to generate the random allocation sequence
"They were randomly assigned to an experiment (N = 35) and a control group (N = 35) using a computerized random number generator."
8b) CONSORT: Type of randomisation; details of any restriction (such as blocking and block size)
"They were randomly assigned to an experiment (N = 35) and a control group (N = 35) using a computerized random number generator."
9) CONSORT: Mechanism used to implement the random allocation sequence (such as sequentially numbered containers), describing any steps taken to conceal the sequence until interventions were assigned

"They were randomly assigned to an experiment (N = 35) and a control group (N = 35) using a computerized random number generator."

10) CONSORT: Who generated the random allocation sequence, who enrolled participants, and who assigned participants to interventions

"They were randomly assigned to an experiment (N = 35) and a control group (N = 35) using a computerized random number generator."

11a) CONSORT: Blinding - If done, who was blinded after assignment to interventions (for example, participants, care providers, those assessing outcomes) and how

11a-i) Specify who was blinded, and who wasn't

"The participants undertook a performance pre-test consisting of a simulation-based assessment which took place at the university simulation laboratory. They hid their identities from the raters by putting on caps, gowns and masks"

11a-ii) Discuss e.g., whether participants knew which intervention was the "intervention of interest" and which one was the "comparator"

Not applicable as only the experimental group received intervention

11b) CONSORT: If relevant, description of the similarity of interventions

Not applicable as the comparator is not a placebo

12a) CONSORT: Statistical methods used to compare groups for primary and secondary outcomes

"Descriptive statistics were computed for the demographic variable and participants' perceptions of the web-based simulation. Differences in demographic characteristics between the two groups were examined by a Chi-square test and t-test. Inter-rater reliability was assessed using an intraclass correlation coefficient (ICC). Changes between baseline and post-test scores were determined by a paired t-test. ANCOVA was performed to evaluate the post-test scores between groups with baseline scores as a covariate. The comments for the two open-ended questions were coded and analyzed for recurring themes."

12a-i) Imputation techniques to deal with attrition / missing values

Not applicable as no missing values was reported

12b) CONSORT: Methods for additional analyses, such as subgroup analyses and adjusted analyses

Not applicable as subgroup analyses was not applied

RESULTS

13a) CONSORT: For each group, the numbers of participants who were randomly assigned, received intended treatment, and were analysed for the primary outcome

"They were randomly assigned to an experiment (N = 35) and a control group (N = 35) using a computerized random number generator. However, three participants from the control group did not turn up for the study, leaving that group with 32 participants"

13b) CONSORT: For each group, losses and exclusions after randomisation, together with reasons

"They were randomly assigned to an experiment (N = 35) and a control group (N = 35) using a computerized random number generator. However, three participants from the control group did not turn up for the study, leaving that group with 32 participants"

13b-i) Attrition diagram

"They were randomly assigned to an experiment (N = 35) and a control group (N = 35) using a computerized random number generator. However, three participants from the control group did not turn up for the study, leaving that group with 32 participants"

14a) CONSORT: Dates defining the periods of recruitment and follow-up

"A prospective, randomized controlled trial with a pre-test post-test design was conducted from the period of November to December 2013."

14a-i) Indicate if critical "secular events" fell into the study period

14b) CONSORT: Why the trial ended or was stopped (early)

Ended after completion of the intervention

15) CONSORT: A table showing baseline demographic and clinical characteristics for each group

Most of the participants were female (97%) and Chinese (62.7%), with an average age of 25.58 years (SD = 3.19). Demographic characteristics including gender (P =1.0), ethnicity (P =0.98), age (P = 0.12), years of working experience (P =0.10), and highest qualification (P = 0.40), were similar for both groups of participants. This supported the randomization and homogeneity of the participants between the groups

15-i) Report demographics associated with digital divide issues

Most of the participants were female (97%) and Chinese (62.7%), with an average age of 25.58 years (SD = 3.19). Demographic characteristics including gender (P =1.0), ethnicity (P =0.98), age (P = 0.12), years of working experience (P =0.10), and highest qualification (P = 0.40), were similar for both groups of participants. This supported the randomization and homogeneity of the participants between the groups

16a) CONSORT: For each group, number of participants (denominator) included in each analysis and whether the analysis was by original assigned groups

16-i) Report multiple "denominators" and provide definitions

Not applicable

16-ii) Primary analysis should be intent-to-treat

17a) CONSORT: For each primary and secondary outcome, results for each group, and the estimated effect size and its precision (such as 95% confidence interval)

"Figure 3 shows that the clinical performance pre-test scores did not differ significantly between the experimental and control groups. Within the groups, the clinical performance post-test scores for the experimental group were significantly higher than the pre-test scores (P < .001). No significant difference was found between the clinical performance pre-test and post-test scores for the control group. Between-group comparisons found significantly higher clinical performance post-test scores (P < .001) for the experimental group than the control group after controlling the pre-test scores. The means scores from the participants' rating (experimental group) on the seven-point scale indicated that that they were highly satisfied with the web-based simulation (mean=6.00, SD=0.79), positive about the quality of the system (mean=6.04, SD=0.68) and information (mean= 5.13, SD=0. 62), and thought highly of the net benefits (mean=6.11, SD=0.71) of the program."

17a-i) Presentation of process outcomes such as metrics of use and intensity of use

No process outcomes defined and measured

17b) CONSORT: For binary outcomes, presentation of both absolute and relative effect sizes is recommended

No binary outcomes were measured.

18) CONSORT: Results of any other analyses performed, including subgroup analyses and adjusted analyses, distinguishing pre-specified from exploratory

Not applicable as not other analysis was performed

18-i) Subgroup analysis of comparing only users

Not applicable as no subgroup analysis was performed

19) CONSORT: All important harms or unintended effects in each group

No harms and intended effects occurrd

19-i) Include privacy breaches, technical problems

Not applicable as there is no occurence

19-ii) Include qualitative feedback from participants or observations from staff/researchers

"Three themes with categories emerged from the written comments on the most valuable aspects of the web-based simulation: relevance to practice as it provides useful information (i.e., “provide specific information which is very useful for us nurses.”) and allows application in a practice setting (i.e., “can apply in real-life situation”); instructional strategies including animation, e-simulation and mnemonics (i.e., “ABCDE and ISBAR” make it simple and clear to understand”) fostering problem solving through knowledge gained (i.e., “understand the signs and symptoms of early deterioration”) and the use of critical thinking (i.e., “utilizes critical thinking to choose appropriate nursing assessment and intervention.”). Three main themes emerged on ways to improve the WBL program; more variation; more information; and, technical features."

DISCUSSION

20) CONSORT: Trial limitations, addressing sources of potential bias, imprecision, multiplicity of analyses

20-i) Typical limitations in ehealth trials

"This study has strengths and limitations. A rigorous research methodology, RCT, was employed to evaluate the learning outcomes. However, the quality of the evidence could be limited by the no-intervention control group. Given that this study is looking at the development of a new WBL program for hospital nurses, the no-intervention controlled study is still considered valuable in the early stages of an innovation. The survey evaluation, particularly the qualitative data, shed light on the value of the program and ways to enhance it. Comparisons of other WBL instructional designs could be conducted in future studies to enhance the program in the future. We tested the learners’ clinical performances using a validated and tested instrument. However, due to logistic constraints, we did not measure the long-term retention of clinical performances which can deteriorate over time."

21) CONSORT: Generalisability (external validity, applicability) of the trial findings

21-i) Generalizability to other populations

Not applicable due to limitations described in 20 (I)

21-ii) Discuss if there were elements in the RCT that would be different in a routine application setting

Not applicable

22) CONSORT: Interpretation consistent with results, balancing benefits and harms, and considering other relevant evidence

22-i) Restate study questions and summarize the answers suggested by the data, starting with primary outcomes and process outcomes (use)

"The randomized controlled study provided evidence on the effectiveness of a web-based simulation in improving hospital nurses’ acute care competencies. The development of these competencies required the hospital nurses to apply and integrate a broad range of knowledge, skills and attitudes. In this study, we developed a web-based simulation that provided opportunities for the learners to work on authentic nursing activities in authentic learning environment. Authentic activities are tasks learners perform in solving real-life problems [24]. An authentic environment resembling a hospital ward was developed to enhance the learning experience. By providing a context that reflects the way knowledge and skills will be used in actual life, the authentic learning environment stimulates the learners to develop competencies relevant for their working lives [25]."

22-ii) Highlight unanswered new questions, suggest future research

Moving forward, future studies could determine the higher-order outcomes of the program on actual clinical practice by evaluating nurse behaviours in practice and their effects on patient care.

Other information

23) CONSORT: Registration number and name of trial registry

This is an educational research not clinical trial

24) CONSORT: Where the full trial protocol can be accessed, if available

Available on request from authors

25) CONSORT: Sources of funding and other support (such as supply of drugs), role of funders

"This study was supported by NUS research start-up grant funded by Yong Loo Lin School of Medicine and Alice Lee Centre for Nursing Studies"

X26-i) Comment on ethics committee approval

"The National University of Singapore (NUS) Institutional Human Research Ethics Board approved the study."

x26-ii) Outline informed consent procedures

All participants were given a participant information sheet that explained the purpose of the study

X26-iii) Safety and security procedures

"All participants were given a participant information sheet that explained the purpose of the study"

X27-i) State the relation of the study team towards the system being evaluated

"The first author is the owner and developer of the software"
